# The effects of monoculture and intercropping on photosynthesis performance correlated with growth of garlic and perennial ryegrass response to different heavy metals

**DOI:** 10.1186/s12870-024-05371-3

**Published:** 2024-07-11

**Authors:** Imran Ali, Javaid Hussain, Benjawan Yanwisetpakdee, Irfana Iqbal, Xiaoming Chen

**Affiliations:** 1 College of Life Science and Engineering, Southwest University of Science and Technology, Mianyang Sichuan, 621010 China; 2 Institute of Molecular Biology and Biotechnology, University of Lahore, Lahore, Pakistan; 3 Institute of Biochemistry, University of Balochistan, 87300 Quetta, Pakistan; 4 Biology Program, Faculty of Science and Technology, Songkhla Rajabhat University, Songkhla, 90000 Thailand

**Keywords:** Biomass, Soil contamination, Phytoremediation, Intercrop, Photosynthetic limitations

## Abstract

**Background:**

The potential of phytoremediation using garlic monoculture (MC) and intercropping (IC) system with perennial ryegrass to enhance the uptake of cadmium (Cd), chromium (Cr), and lead (Pb) were investigated.

**Results:**

Positive correlations were found between MC and IC systems, with varying biomass. Production of perennial ryegrass was affected differently depending on the type of toxic metal present in the soil. Root growth inhibition was more affected than shoot growth inhibition. The total biomass of shoot and root in IC was higher than MC, increasing approximately 3.7 and 2.9 fold compared to MC, attributed to advantages in root IC crop systems. Photosystem II efficiency showed less sensitivity to metal toxicity compared to the control, with a decrease between 10.07–12.03%. Among gas exchange parameters, only Cr significantly affected physiological responses by reducing transpiration by 69.24%, likely due to leaf chlorosis and necrosis.

**Conclusion:**

This study exhibited the potential of garlic MC and IC with perennial ryegrass in phytoremediation. Although the different metals affect plant growth differently, IC showed advantages over MC in term biomass production.

## Introduction

Metals come into the soil environment from different sources, including natural and volcanic eruptions, forest fires, evaporation from the surface of the oceans and the development of civil urbanisation and industrialisation activities [1]. Heavy metal pollution associated with the soil may have different forms, having a remarkable ability to form soluble salts and oxides, which do not go decomposition or cannot be destroyed biologically, and they can transform from one oxidation state or to other organic compounds [2]. Fuel and energy production, mining and smelting of metals, fertiliser, gas exhaust, sewage, electroplating, and pesticide application have dramatically increased soil contamination [3, 4]. Heavy metals are hazards to the ecosystem through the food chain, direct ingestion and contact with soil contamination [5, 6].

There are two kinds: essential micronutrients for average plant growth (Fe, Mn, Zn, Cu) and non-essential heavy metals with unknown physiological and biological functions [7]. Non-essential and very toxic even in minute quantities of heavy metals such as Cd, Cr and Pb. Cr high level is toxic and causes human allergic dermatitis and is associated with carcinogenic [8, 9]. Cd is highly toxic to humans and animals. Increased accumulation of Cd forced renal dysfunction, reproductive organs, liver, kidney damage, and pulmonary emphysema diseases [10, 11]. Soil contamination with Pb decreases soil fertility, affecting plant growth and crop production. High level of Pb contributes to brain damage, headache, central nervous system disorder, colic and anemia. Pb poisoning causes oxidative stress, disrupting the delicate antioxidant balance of mammalian cells and causing severe injury to the red blood cells and kidney [7, 12].

There are different methods to remediate the soil ecosystem and restoration, whereas phytoremediation technology is environment-friendly and cost effective, eliminating toxic metals from the soil [13]. Plants including grasses, shrubs, and trees absorb and volatilize heavy metals from polluted soil, some can accumulate As, Pb, Hg, Cr, Zn, Cu and get trapped in roots [14]. Accumulation of heavy metals in plant harvestable tissues involves the metals-tolerant to extract contaminates from the soil; however, phytoremediation efficiency depends upon heavy metals toxicity, slow growth and low biomass [15, 16]. The application of using inorganic and organic soil amendments improves growth [17] and productivity by reducing the mobility of toxic heavy metals such as Cd, Pb, and Ni in soil and subsequently in various parts of plants. Moreover, inorganic and organic amendments can serve as a crucial strategy for improving soil quality and sustainable management by immobilizing heavy metals and reducing their accumulation in plant tissues [18, 19].

Heavy metals stress affects the production and some other aspects of the plant biochemistry and physiology [20]. Excess uptake of metals begins to produce cytotoxicity, imbalance in nutrients and pH, interfering in cellular processes and physiological effects in plants. The physiology and composition of roots, including their lipids, play a crucial role in the uptake of phytotoxic chemicals [21]. High levels of metals in soil can reduce the water uptake by roots, causing osmotic effects [22], promoting cellular turgor decrease and leaf stomatal closure, promoting cell biophysics regulated by plants to avoid water losses due to evapotranspiration, as well as root system to approach and absorb accessible water, conserving the inner water balance [23]. Photosynthesis uptake is limited by CO_2_ availability as substrate, reducing stomatal conductance [24]. Combining CO_2_ fixation and estimating photosystem II (PSII) activity in chloroplast energy management during stress can reflect plant health state [25]. PSII can be destabilized by excessive radiation promoting photodamage and electron transport activity [26].

Soil structure and ecology can be highly affected by the exudates secreted by the rooting system of garlic (*Allium sativum* L.), as well as allicin, which is a main antimicrobial component of garlic. Interplant of garlic showed the promotion of seedling growth and seed germination. It significantly impacts plant quality, fruit yield, growth pattern and development. Allelochemicals are considered to have significant effects on enzyme function, cell division, photosynthesis, water uptake and metabolism [27].

Interplant is a crop management system which protects the soil structure and physiochemical properties and provides a better option to avoid the deleterious effects and accumulation of toxic components [28]. Inter-plantation is also beneficial for soil erosion control, soil fertility pest or disease control due to less proportion of weeds, which reduces crop loss and the leaching of nutrients. It can increase the amount of nitrogen in the soil [29].

The plant species capable of accumulating heavy metals has investigated. Consequently, the quest for plant proficient in extracting heavy metals efficiently necessitates traits such as growing fast, environmental adaptability, and compatibility for intercropping with other species. Ryegrass (*Lolium perenne* L.) is used as a model plant, exhibits rapid growth and displays swift inhibition of root growth in response to toxic concentrations of heavy metals, making it an ideal candidate for studying tolerance across diverse environments and soil types, particularly in phytoremediation research. Its frequent utilization in the restoration of tailings sites, achieved through iterative sowing and harvesting, renders it well-suited for remediating soils contaminated with heavy metals [13].

The present study investigates garlic's performance and effects for removing heavy metals and the characteristics, distribution, and mechanisms of different species in cultivation systems (monoculture and intercrop). It has been recognized for its capacity to withstand both biotic and abiotic environmental stresses, such as bacterial, viral, and oxidative stresses. It also exhibits potential resilience against certain heavy metals, as an intercrop plant with hyper-accumulators [30]. The results were correlated with the photosynthetic pigments, chlorophyll fluorescence, gas exchange analysis and the effects of garlic intercropped with Perennial ryegrass. 

## Materials and methods

### Experiment design and plant materials

In 2018, the soil was collected at Southwest University of Science and Technology (104^o^41′37″, 31^o^32′18) at Mianyang, Sichuan Province of China. The collected soil was air dried at ambient room temperature for several days and passed through a 2-mm sieve for general analysis. The soil is a meadow with a pH of 7.86, EC of 4.7 mS/cm, moisture content of 26.24%, carbon content of 127.33 g/kg^−1^ and organic matter of 13 g kg^−1^. Dry soil, weighing 1 kg each, was placed in zip locked plastic bags and treated with three levels of each heavy metal along with a control. All pots, arranged in a randomised complete-block design, were randomly grouped into two for the treatments. Pots experimented with three replicates, which were placed in an open shed greenhouse condition. Relative humidity was 70%, the average temperature was 6–16 °C, and the warmest month, with a temperature of 26 °C. The treatments including the following (1) Perennial ryegrass Monoculture (MC) (2) Perennial ryegrass with Garlic intercropping (IC) were used in this research. Mature and healthy equal size of Garlic seedlings from covers were separated and developed in distilled water for one week with slight changes [30]. Perennial ryegrass seeds were washed with tap water and two times deionised water. Seeds surface were disinfected with 0.5% NaClO solution (5 min, 26 °C) followed thoroughly washed with distilled water. The seeds were germinated on a filter paper soaked with 10 mL of deionised water and placed in a dark place at room temperature (25 °C), germination occurred in three days. Seedlings were planted with three replicates contaminated soils pots with different concentrations slight changing [31]. Plants were irrigated with deionised water every alternate day. After sixty days of sowing plants were harvested and determined for different parameters [30, 31].

Collected soil was supplemented with Cadmium Chloride CdCl_2_.2H_2_O, 0.3 mgkg^−1^ 0.9 mgkg^−1^, other treatment Chromium (III) Chloride hexahydrate (CrCl_3._6H_2_O) concentration was 150 mgkg^−1^ 350 mgkg^−1^ and Lead nitrate Pb (NO_3_)_2_ 100 mg/kg^−1^ and 300 mgkg^−1^ with three replicates each, respectively.

### Biomass and growth comparison

After sixty days of heavy metal treatments, plants were harvested with good care and washed with tap water several times, followed by distilled water to remove soils. Plants were separated into shoots and roots, and their fresh weight (FW) was weighed immediately. The Number of leaves, roots, plant height, and root length (cm) was calculated. For the measurement of dry matter content, fresh plant tissues were dried at 60 ° C for 72 h. The water content of plant biomass fresh leaves and roots were taken an oven at 65 ° C for 48 h and immediately weighted the obtained values expressed according to (FW-DW) × 100/FW.

An additional set of plant tissues was collected and processed with liquid nitrogen and then stored at -80 ° C for further biochemical analysis.

### Photosynthesis, leaf gas exchange, stomatal conductance, chlorophyll a, PSII and metal analysis

Determinations were analysed after sixty days for all treatments. Open-type portable measurement system (Li-6400–40 leaf chamber fluorometer Li-Cor Inc. Japan). The determination setting was maintained at 25 ° C. Cavetti measurement CO_2_ concentration was set at 400 ppm, and relative humidity was maintained at around 55%. Fully expanded and healthy leaves with similar exposition to radiation were used for all plants. CO_2_ net assimilation value rate (A), transpiration rate (E), stomatal conductance (gs) and intercellular CO_2_ concentration (Ci) were analysed [32].

The possible efficiency of PSII photochemistry (Fv/Fm) was calculated. The leaves were adapted on dark conditions before analysis as Fv/Fm = (Fm-Fo)/Fm, Fm and Fo are the maximum and minimum fluorescence yield emitted by the leaves in the dark-adapted state respectively. The real photochemical efficiency of PSII in the light was analysed as ΦPSII = (Fmʹ-Fʹ)/Fmʹ [33], where Fmʹ is the maximum fluorescence yield with all PSII reaction centres in the reduced stat obtained superimposing.

Non-photochemical fluorescence quenching (NPQ) was analysed according to the stern–volmer equation as NPQ-(Fm/Fmʹ)-1 [34].

Dry pot soil samples were grounded using an agate mortar and digested into boiling aqua regia for 2 h. The digested sample were adjusted to 50 ml with distilled water. Heavy metals analysis was done by using the Perkin Elmer AAS [35, 36]. Bioavailable heavy metals in the soil were extracted using EDTA [36]. Dry plant samples were grounded in to a power and digested in a mixture of HNO_3_ and HClO_4_ (4:1, v/v). Heavy metals concentration in the digested sample was determined by ICP analysis.

### Statistical analysis

Pots were arranged in a randomised complete-block design with three replicates for each treatment and one control. Using PASW Statistics 18 SPSS software applied for the data were subjected to analysis of variance (ANOVA), means comparison were performed using least significant difference (LSD) at *P* < 0.05 multiple range test to determine the relationships between the different treatments based on biometric and physiological parameters, data recorded at the end of the experiment.

## Results

### Plant biomass and development of shoots and roots

Heavy metals toxicity in soil affected the growth performance, resulting in the total biomass of IC being much higher than that in the MC plants. Although the control showed lower biomass yield than the toxicity of the metal, the total biomass in control and Cr treatment of IC increased approximately 3.7 and 2.9 fold compared to MC crop. Meanwhile, the total biomass of the control, Cd0.3 and Pb300, was slightly lower in the IC than in the MC crop, which exhibited the differences in the effects of metal treatment.

**Fig. 1:**
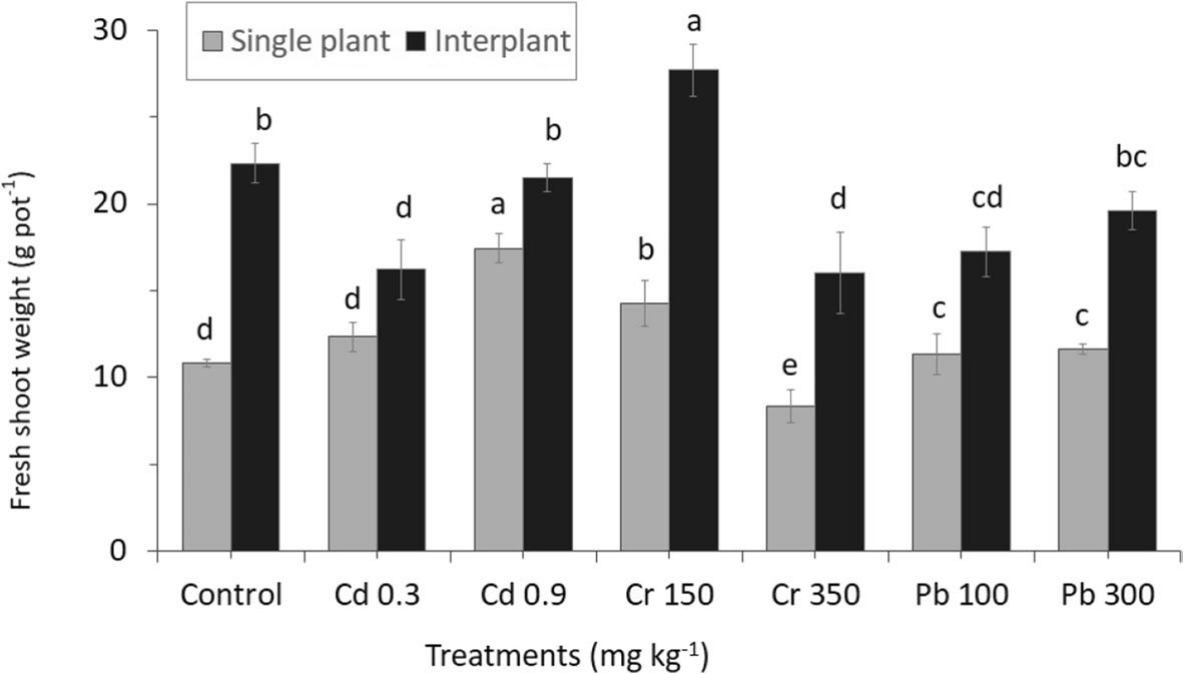
Effect of different concentrations of heavy metals toxicity on plant biomass shows fresh shoot weight of perennial ryegrass in MC and IC grown in nutrients soil for 60 days. The letters for each cropping method are separated. Error bars indicate standard error (SE) of the mean (*n* = 3)

**Fig. 2:**
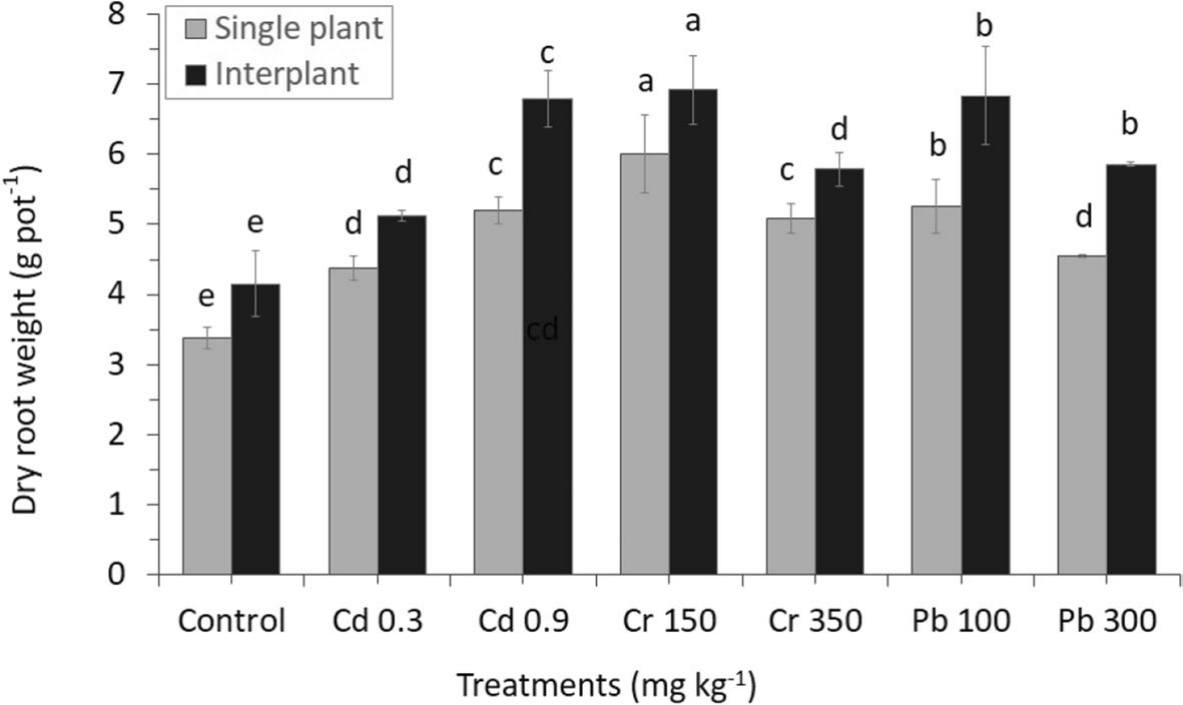
Effect of different concentrations of heavy metals toxicity on plant biomass shows root dry weight of perennial ryegrass in MC and IC grown in nutrients soil for 60 days. The letters for each cropping method are separated. Error bars indicate standard error (SE) of the mean (*n* = 3)

The toxicity in soil changed the biomass yield of shoot and root in all treatments. Cr and Pb toxicity reduced DW of whole plants (except for Cd), with higher concentrations (Figs. 1 and 2). This was indicated by a huge reduction in plant growth, which was detected in Cr350 in comparison with Pb, which is slower to decrease. However, the Cd treatments and total biomass in shoot and root were significantly increased than in the control.

**Fig. 3:**
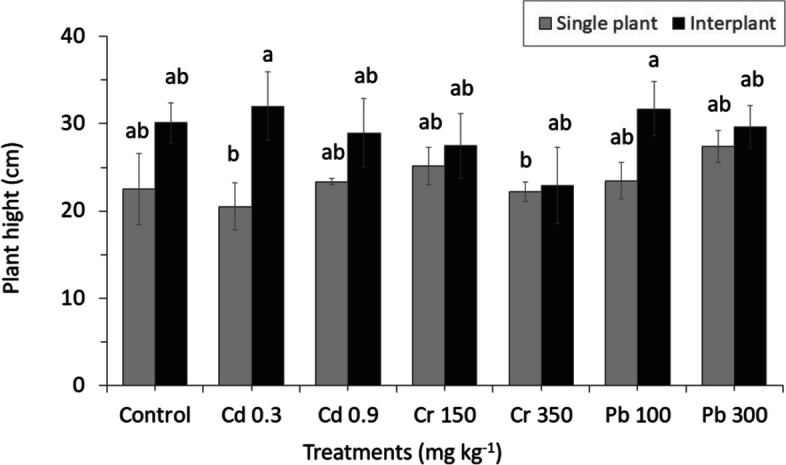
The result of different concentrations of heavy metals on the growth of shoots between MC and IC. The letters for each cropping method are separated. Error bars indicate standard error (SE) of the mean (*n* = 3)

Shoot height was found to be less sensitive to heavy metals than root length, and the level of metal concentration showed significant differences between MC and IC crop systems (Fig. 3). Although the shoot growth of IC in all treatments was decreased at high concentrations, the growth rate of MC plant was increased (except for Cd). The low concentration affected the shoot growth of IC, and the highest growth was found in Cd03 and Pb100. In contrast, a high concentration of Cr350 reduced shoot growth.

**Fig. 4:**
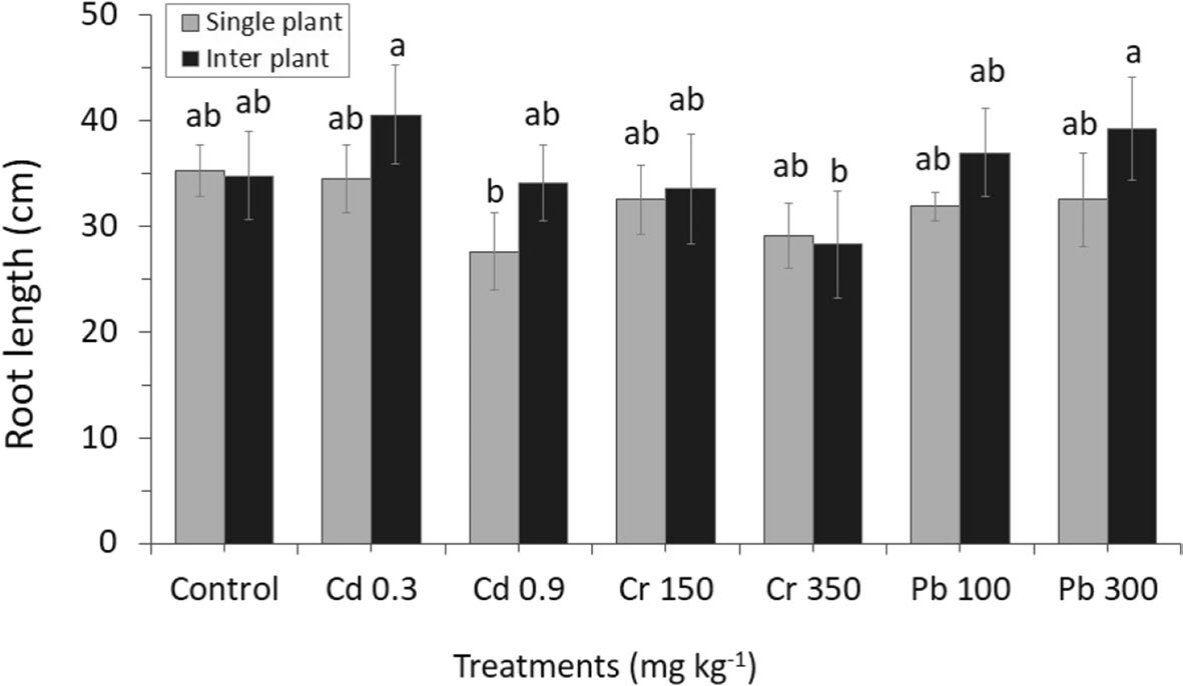
The result of different concentrations of heavy metals on the growth of roots between MC and IC. The letters for each cropping method are separated. Error bars indicate standard error (SE) of the mean (*n* = 3)

Root growth was more sensitive to metal toxicity than the shoot. Although no difference of root length was found in the control group, the high concentration of Cd and Cr seemed to affect the root, and decreased the root length both in MC and IC. On the other hand, at high concentration of Pb increased the root length of MC and IC crops (Fig. 4).

**Fig. 5:**
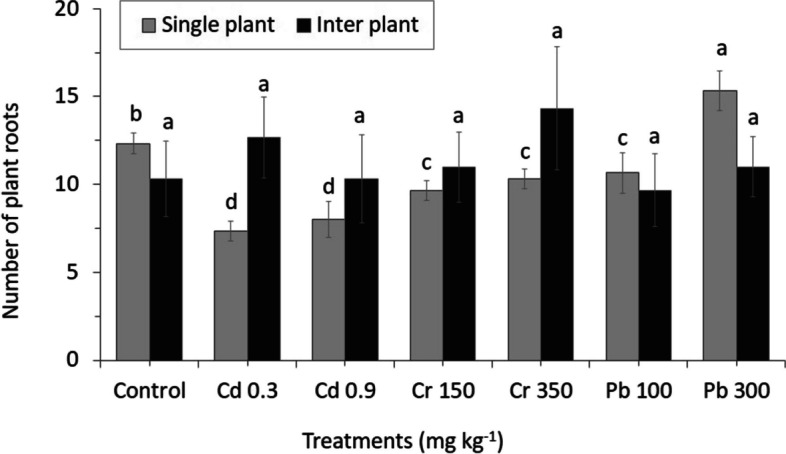
The number of roots from perennial ryegrass grown in different concentrations of heavy metals between MC and IC crop. The letters for each cropping method are separated. Error bars indicate standard error (SE) of the mean (*n* = 3)

**Fig. 6:**
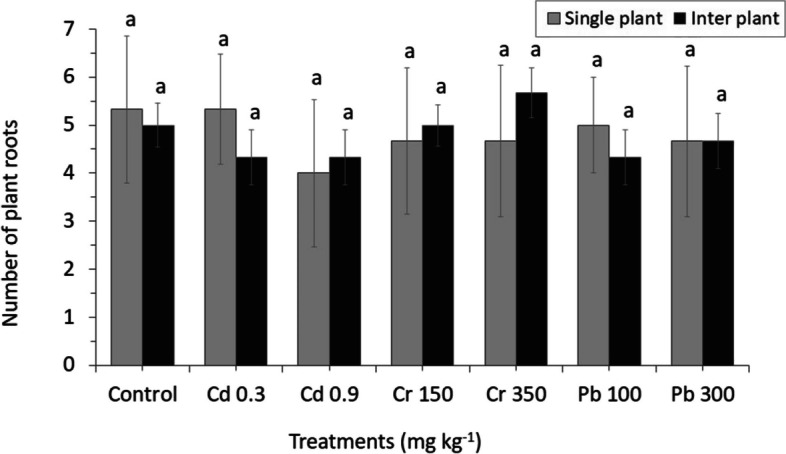
The number of leaves from perennial ryegrass grown in different concentrations of heavy metals between MC and IC crop. The letters for each cropping method are separated. Error bars indicate standard error (SE) of the mean (*n* = 3)

The diverse effect in root number with different concentration of heavy metals was found between MC and IC (Fig. 5). The root number of all treatments in MC crop was lower than the control (except for Pb300) and significantly increased with the concentration that was similar to the IC (except for Cd). In addition, there was no difference in the effect of toxicity from heavy metals to the number of leaves; even general chlorosis of healthy and young leaves appeared in all heavy metal treatments (Fig. 6).

### Chlorophyll a/b fluorescence and influence of photosynthetic rate

**Fig. 7:**
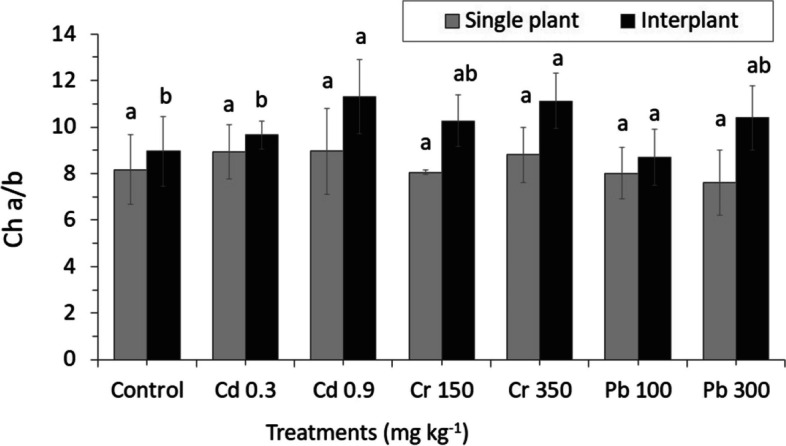
Changing of Photosynthetic pigments in leaves of MC and IC with garlic subjected to different concentrations of heavy metals after 45 days of metals stress. The letters for each cropping method are separated. Standard errors (SE) of the mean (*n* = 3) with LSD (*p* < 0.05)

Photosynthetic pigment changes in the leaf were observed, both in MC and IC ryegrass with garlic as shown. According to the metal contents of control, which in the MC plant was lesser than IC, the leaves chlorophyll ratio was increased in both treatments MC and IC with increasing the concentration of heavy metals (Fig. 7). Compared to control, Fv/Fm of ryegrass MC was significantly affected by different heavy metal treatments, while IC Fv/Fm significantly and slightly increased both in control and other heavy metal treatments.

**Fig. 8:**
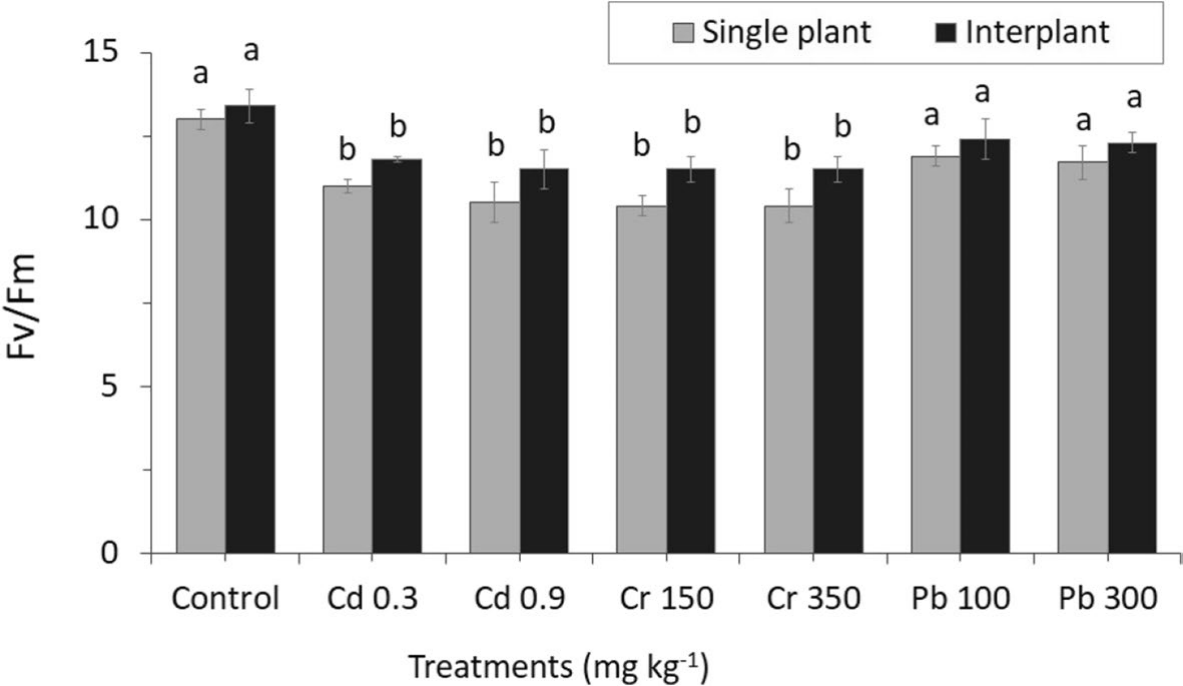
Changing of Leaf fluorescence parameters of ryegrass and effects of garlic on the maximum quantum efficiency of the PSII (Fv/Fm). The letters for each cropping method are separated. Standard errors (SE) of the mean (*n* = 3) with LSD (*p* < 0.05)

A decrease in photosynthetic energy conversion efficiency can be seen in all treatments, approximately 10.07- 11.03 and 11.40—12.03% of Cr 350 and Pb 100 for MC and IC, respectively (Fig. 8). Although a marked difference was found to be affected in MC plant than IC when treated with different concentrations, slight differences were found between MC and IC of control.

**Fig. 9:**
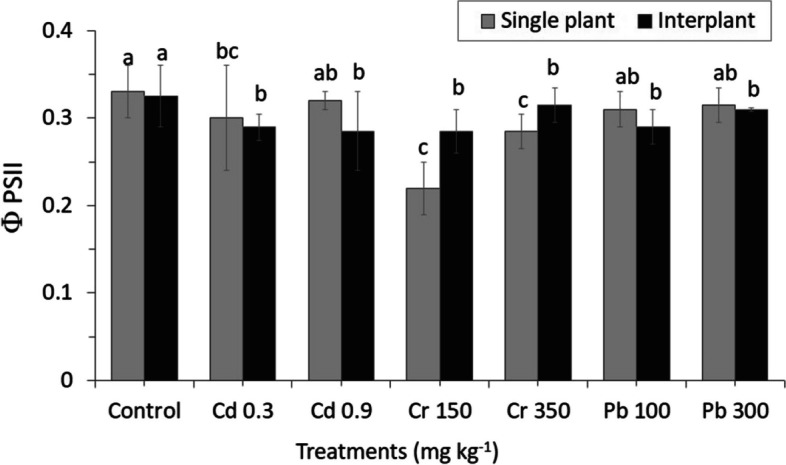
Results of photosynthesis efficiency, actual Photochemical efficiency of photosystem II in the light (ΦPSII). The letters for each cropping method are separated. Standard errors (SE) of the mean (*n* = 3) with LSD (*p* < 0.05)

**Fig. 10:**
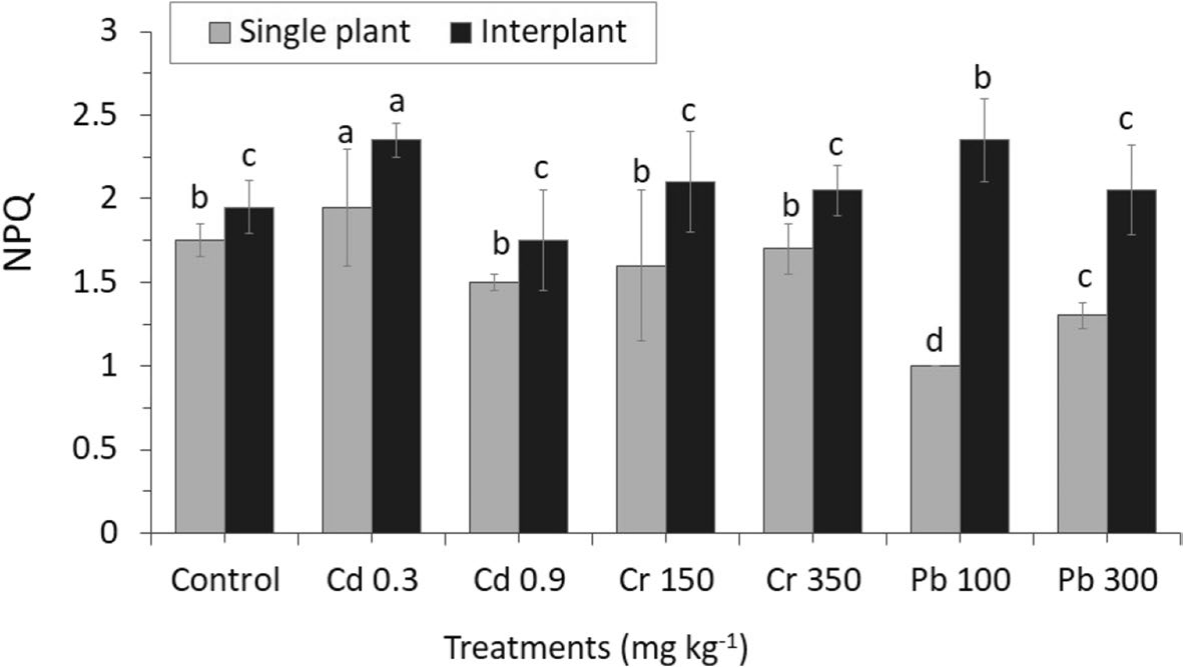
Results of photosynthesis efficiency, non-photochemical florescence quenching coefficient (NPQ). The letters for each cropping method are separated. Standard errors (SE) of the mean (*n* = 3) with LSD (*p* < 0.05)

ΦPSII was not significantly affected by heavy metal concentrations. Control plants ΦPSII values were slightly similar and showed the highest rate both in MC and IC. On the other hand, ΦPSII Cr decreased significantly in MC while increasing significantly in IC treatments (Fig. 9). Heavy metals stress also affected the non-radioactive energy dissipation capacity both in MC and IC, as shown by increasing NPQ, maintaining intermediate value, and slightly reducing Pb treatments. NPQ reached the highest value at Pb 100 2.395 ± 0.306b in IC, and the reduced value was observed in the range of 1.06 ± 0.048d in MC Pb 100 (Fig. 10).

### Photosynthesis, leaf gas exchange, stomatal conductance and PSII

**Fig. 11:**
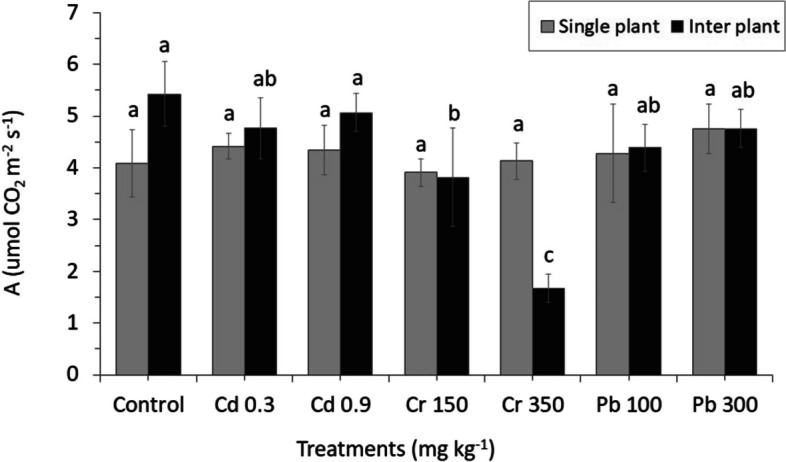
Net CO_2_ assimilation rate (A). The letters for each cropping method are separated. Error bars indicate standard error (SE) of the mean (*n* = 3)

**Fig. 12:**
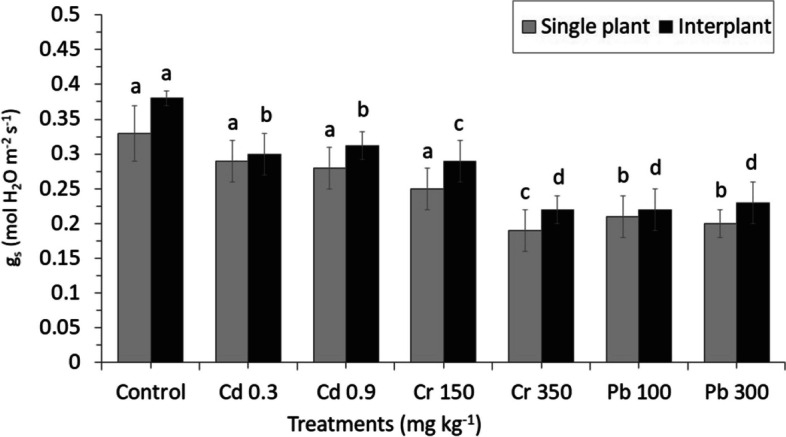
Stomatal conductance (gs). The letters for each cropping method are separated. Error bars indicate standard error (SE) of the mean (*n* = 3)

**Fig. 13:**
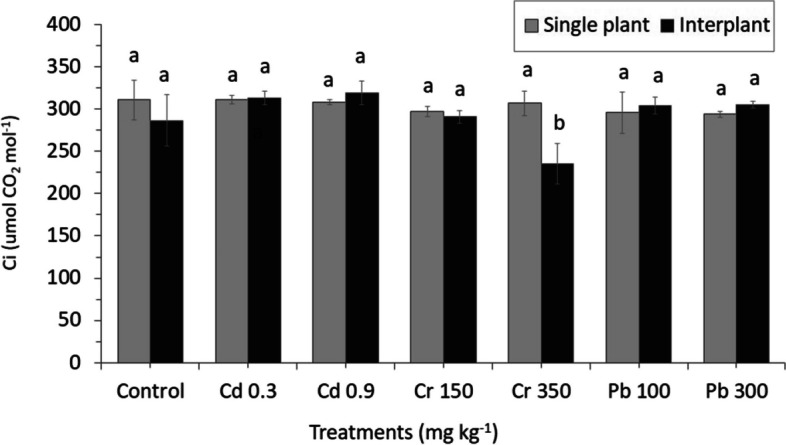
Intracellular CO_2_ concentration (Ci). The letters for each cropping method are separated. Error bars indicate standard error (SE) of the mean (*n* = 3)

**Fig. 14:**
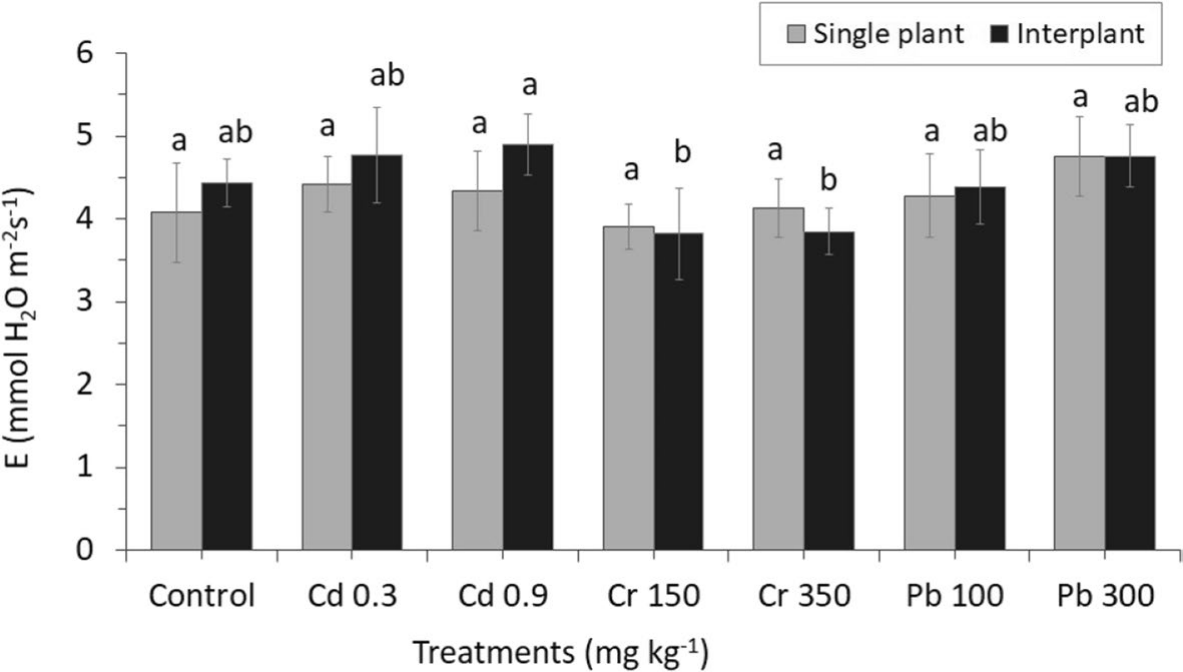
Transpiration rate (E). The letters for each cropping method are separated. Error bars indicate standard error (SE) of the mean (*n* = 3)

Heavy metals applied in the experiments produced only a few significant adverse effects on the physiological response of MC and IC crops. There were no different effects on the MC ryegrass for the parameters assimilation rate (A), stomatal conductance (gs) and intercellular CO_2_ concentration (Ci) (Figs. 11, 12 and 13). Similar result was also found for the transpiration rate (E) parameter of IC. Among physiological changes of MC plant, only parameter transpiration rate (E) significantly decreased, whereas Pb treatment showed a significant increased from 12.013 E (mmol H_2_O m^−2^ s^−1^) in Pb100 to 12.032 E (mmol H_2_O m^−2^ s^−1^) in Pb300 by increasing the levels of Pb in the crop (Fig. 14).

A high concentration of Cr350 was found to be affected on the gas exchange parameters (Ci, A, and gs) of IC; only Ci significantly decreased from 290.66 Ci (µmol CO_2_ mol^−1^) in Cr150 to 235.33 Ci (µmol CO_2_ mol^−1^) in Cr350 by increasing the concentration (Fig. 13). Similar result was also found in parameter A which decreased from 3.82 A (µmol CO_2_ m^−2^ s^−1^) in Cr150 to 1.67 A (µmol CO_2_ m^−2^ s^−1^) in Cr350, whereas a different result was found in Cd increased from 4.77 A (µmol CO_2_ m^−2^ s^−1^) in Cd 0.3 to 5.07 A (µmol CO_2_ m^−2^ s^−1^) in Cd 0.9 by increasing the concentration. The stomatal conductance was found to be more sensitive to heavy metals than the other gas exchange parameters, the reduction was detected in all treatments, especially in Cr, which significantly decreased than in the control (Fig. 12).

### Uptake of heavy metals, tolerance and translocation

The total Cd and Pb contents in plant roots significantly increased with the increasing of concentration, and the same result was found in plant shoots, both in MC and IC (Table 1). In addition, the total Cr content from root of IC was also the same. However, the MC plant exhibited the result that Cr content was significantly higher in Cr150 than in Cr350, nearly 4 times higher than that in the Cr350. It should be noted that the Cr content in the root of IC was over 7 times higher than the control when the concentration was increased.

**Table 1:** Concentration of Cd, Pb and Cr in roots and shoot of perennial ryegrass MC, and IC with garlic (mg/kg)

Metals treatments (mg/kg)	Cd Root MC	Cd Root IC	Cd Shoot MC	Cd Shoot IC
Control of Cd	0.20 ± 0.05	0.21 ± 0.03	0.48 ± 0.09	0.65 ± 0.01
Cd 0.3	1.54 ± 0.19	1.64 ± 0.11	0.61 ± 0.02	0.71 ± 0.04
Cd 0.9	2.38 ± 0.38	2.39 ± 0.35	0.97 ± 0.06	0.98 ± 0.019
Metals treatments (mg/kg)	Pb Root MC	Pb Root IC	Pb Shoot MC	Pb Shoot IC
Control of Pb	5.27 ± 0.34	5.34 ± 0.35	1.68 ± 0.20	1.97 ± 0.10
Pb100	17.92 ± 1.60	39.23 ± 3.71	11.92 ± 0.83	13.20 ± 0.87
Pb 300	54.15 ± 0.92	61.31 ± 2.77	15.72 ± 2.18	17.81 ± 0.08
Metals treatments (mg/kg)	Cr Root MC	Cr Root IC	Cr Shoot MC	Cr Shoot IC
Control of Cr	11.39 ± 1.38	22.76 ± 0.53	6.04 ± 0.35	10.49 ± 0.20
Cr 150	17.72 ± 5.43	28.36 ± 4.80	31.66 ± 18.64	74.03 ± 13.11
Cr 350	19.43 ± 3.99	29.31 ± 0.47	43.66 ± 0.35	68.85 ± 4.47

## Discussion

The evaluation of MC and IC efficiency can be elucidated by analysing the results of heavy metal extraction and biomass production. In the present study, IC is shown to increase concentrations of Cd, Cr and Pb in plant organs while also contributing to total biomass. The heavy metals (Cd, Cr and Pb) in soil from the experiment affected the biomass yield, which was independent of the concentration of heavy metals and varied among different crop systems and plant species [35]. Generally, Cd, Cr and Pb do not function as nutrients in plants and seem highly toxic to many organisms in the ecosystem, including microorganisms and plants [36]. Due to the content of Cd, Cr and Pb found in plants were 0.1—2.4, 0.006—18 and 2—3 mg^•^kg ^−1^, respectively [37]. Cd contents in this study were at normal levels; the result showed no effect with plant growth in Cd treatments [38]. However, the high concentration of Cr and Pb can be harmful and toxic to plant growth, resulting in decreased plant biomass, in a consequence of severe phytotoxicity and reduced photosynthetic activity [39, 40]. It has been reported that toxicity inhibited root growth of *Lolium perenne* in the following order: lead > cadmium > chromium [41]. Morphological studies of ryegrass reveal a porous structure that is highly conductive to capturing and immobilizing heavy metals within these pores [13].

The limiting factors of perennial ryegrass production depend on the different types of toxic metals and the quantity detected in the soil. Although the toxicity of Cd, Cr and Pb inhibited shoot and root growth, the shoot was found to be less harmful to heavy metals than root, while the result varied from roots and leaves. This may be because the root could play an important role by preventing excess toxicity accumulation and protecting the shoot part against heavy metal translocation [42]. Moreover, rhizosphere microbes play a vital role in enhancing soil heavy metal availability in plant cells by transforming metals into bioavailable and soluble forms through siderophores, organic acids, biosurfactants, biomethylation, and redox processes [35, 43]. Symptoms from heavy metals appeared in shoots and leaves, by reduction of shoot height, then became chlorosis that may occur by root damage from toxicity in soil. It has been suggested that these symptoms lie in the roots because plants are unable to utilize iron, resulting in an iron deficiency and suppressed root and shoot elongation [44].

The results revealed a higher amount of Cd, Cr, and Pb detected in both shoots and roots in IC compared to the MC, suggesting better uptake, translocation and mobilization within soil in the IC system. In general, the solubility of heavy metals in soils is regulated by pH, the amount of cations exchange capacity, the content of organic carbon, and the oxidation state of the system [45]. In addition, the monocot root system pursued a somewhat zigzag course through the soil and the garlic root system spread at a depth between 2.5–5 inches in the early stage and maximised of 11 inches were attained, while a ryegrass root grew at 10–25 inches in depth. The results from this study exhibited that the biomass of IC was higher than the MC in shoots and roots. That is because the different root IC crop system levels provided the advantage of the association between ryegrass and garlic. In this experiment, IC systems engender the competition among perennial ryegrass and garlic, demonstrate that perennial ryegrass exhibited greater biomass compared to those in MC, thereby indicating the more competitive prowess than garlic [46]. Additionally, plants in the IC system were taller than in the MC, thereby possessing advantages in light, resulting in higher yields.

The available resources, nutrients and soil spaces between IC crop systems increased productivity by potentially compensating for environmental growth [47, 48]. Furthermore, the spice nature of garlic makes it a preferred option as a co-culture plant for remediation. This is attribute to the large spectrum of substances that garlic produces and secretes, which can influence the activities of soil microorganisms, supporting beneficial symbioses, alter the chemical and physical properties of the soil, and inhibit the growth of competing plant species by allelopathy [49]. However IC did not significantly affect the accumulation and removal of heavy metals in contaminated soil compared to the MC system [50].

Among gas exchange parameters (A, E, gs and C_i_), only Cr significantly negative effects on physiological response by decreasing the transpiration rate (E) that may be caused by induction of leaf chlorosis and necrosis besides physiological and biochemical alterations [51, 52]. This study observed reduced values of photosynthetic efficiency and photosynthetic pigments of perennial ryegrass after exposure to Cd, Cr, and Pb. The reduction of photosystem II efficiency (Fv/Fm) showed less sensitivity to metals toxicity than control in MC and IC. The result demonstrated that the chloroplast content and photosynthetic potential of IC were higher than the MC. This result could be associated with the content of metals in the biosynthetic process controlled by each metal, which affected the photosynthetic mechanism; as a result, the chlorophyll structure was damaged and lost its function [53]. The reduction of pigment content depends on metal concentration that results in decreasing of chlorophyllase and 5-aminolevulinic acid dehydratase (ALAD) in chlorophyll production [54, 55].

The results exhibited that most treatments accumulated higher concentrations of Cd, Cr, and Pb than the control. It has been reported that the average metal accumulation in rice roots was much higher than in stems and leaves and rice grains [56]. The present study confirmed that the accumulated metals in the root were higher than the shoot for both soil crops treated, and the combinations of IC could enhance the heavy metals concentrations [57]. The differences in the absorption of metals may be due to soil characteristics, including interaction with organic matter, the elements, and chemical immobilisation, among other factors [15] that were not in the scope of this study. Based on the results from this study, IC of ryegrass and garlic has the highest metal concentration, indicating the efficiency in accumulating Cr > Pb > Cd and can be used for phytoremediation.

## Conclusion

The present studies showed that garlic cultivation in IC with other plants such as Perennial ryegrass can positively impact the bioremediation of heavy metals in contaminated soils and the interspecific underground interactions leading to the nutrient providing ability that increases crop yield. The intercropping of garlic with Perennial ryegrass resulted in higher weight, number of leaves, plant height, and root length that proposed IC system can help the plants overcome heavy metal stress. This study suggests that the Perennial ryegrass IC with garlic has a great potential to accumulate heavy metals in its underground parts, particularly roots. The results of the above compositional structure showed no significant difference between the dominant flora in the cultivated ryegrass and ryegrass with garlic IC. We concluded that garlic caused the most significant change in the intercropping system to establish an approach to improve hyperaccumulation of heavy metals Cr, Cd and Pb removal from polluted soil.

## Data Availability

The datasets used and analysed during the current study are available from the corresponding author on reasonable request.
